# Concomitant Autoimmunity in Endometriosis Impairs Endometrium–Embryo Crosstalk at the Implantation Site: A Multicenter Case-Control Study

**DOI:** 10.3390/jcm12103557

**Published:** 2023-05-19

**Authors:** Noemi Salmeri, Gianluca Gennarelli, Valeria Stella Vanni, Stefano Ferrari, Alessandro Ruffa, Patrizia Rovere-Querini, Luca Pagliardini, Massimo Candiani, Enrico Papaleo

**Affiliations:** 1 Gynecology/Obstetrics Unit, IRCCS San Raffaele Scientific Institute, 20132 Milan, Italy; salmeri.noemi@hsr.it (N.S.);; 2 Obstetrics and Gynecology, Physiopathology of Reproduction and IVF Unit, Department of Surgical Sciences, Sant’Anna Hospital, University of Torino, 10124 Turin, Italy; 3 Division of Immunology, Transplantation, and Infectious Diseases, IRCCS San Raffaele Scientific Institute, 20132 Milan, Italy; 4 Department of Brain and Behavioral Sciences, University of Pavia, 27100 Pavia, Italy

**Keywords:** endometriosis, autoimmunity, autoantibodies, implantation, embryo development, pregnancy rate, reproductive endocrinology

## Abstract

Endometriosis and autoimmune diseases share a hyper-inflammatory state that might negatively impact the embryo–endometrium crosstalk. Inflammatory and immune deregulatory mechanisms have been shown to impair both endometrial receptivity and embryo competence at the implantation site. The aim of this study was to investigate the potential additional impact of co-existing autoimmunity in women affected by endometriosis on the early stages of reproduction. This was a retrospective, multicenter case-control study enrolling N = 600 women with endometriosis who underwent in vitro fertilization–embryo transfer cycles between 2007 and 2021. Cases were women with endometriosis and concomitant autoimmunity matched based on age and body mass index to controls with endometriosis only in a 1:3 ratio. The primary outcome was the cumulative clinical pregnancy rate (cCPR). The study found significantly lower cleavage (*p* = 0.042) and implantation (*p* = 0.029) rates among cases. Autoimmunity (*p* = 0.018), age (*p* = 0.007), and expected poor response (*p* = 0.014) were significant negative predictors of cCPR, with an adjusted odds ratio of 0.54 (95% CI, 0.33–0.90) for autoimmunity. These results suggest that the presence of concomitant autoimmunity in endometriosis has a significant additive negative impact on embryo implantation. This effect might be due to several immunological and inflammatory mechanisms that interfere with both endometrial receptivity and embryo development and deserves further consideration.

## 1. Introduction

Endometriosis is an inflammatory, estrogen-dependent, chronic gynecological disorder characterized by the presence of endometrial-like tissue outside the uterus [1]. Immune dysregulation is thought to play a crucial role in its highly complex etiopathogenesis [2]. Indeed, chronic inflammatory response to the ectopic endometrium exacerbates abnormalities in both the cell-mediated and humoral immune systems of women with endometriosis [3]. Consistent with the theory that endometriosis is an immune-related disorder, a direct link between endometriosis and several systemic autoimmune diseases has already been proposed [4,5].

Infertility is one of the main challenges faced by women with endometriosis during their reproductive lifespan [6]. Increasing evidence suggests that immune phenomena are key factors in endometriosis-related infertility [7]. For instance, higher levels of pro-inflammatory cytokines, such as interleukin (IL)-1, 6, 8, and 10, tumor necrosis factor (TNF)-alpha, and vascular endothelial growth factor (VEGF), have been detected in the peritoneal fluid of infertile women with endometriosis [8]. Elevated levels of these cytokines in the peritoneal and follicular fluids (FF) of patients with endometriosis have also been linked to reduced quality of oocytes, altered embryo development, and impaired embryo implantation [9,10,11]. 

The success of implantation depends on a competent embryo, a receptive endometrium, and an effective crosstalk between the two [12]. The immune system at the maternal–fetal interface plays a role in fine-tuning this process so that either excessive immune inhibition or immune hyperactivity is thought to result in implantation failure [13]. In fact, the onset or activity of autoimmune diseases can impair embryo implantation [14]. Firstly, autoimmunity is known to interfere with the maternal endometrial immune-receptive profile by creating an abnormal inflammatory microenvironment at the implantation site [15]. Furthermore, recent findings have shown that autoimmune diseases can directly affect fertilization and cleavage rates, as well as the proportion of good-quality embryos [16,17]. Taken together, these findings suggest that autoimmunity may have a negative impact not only on the endometrium but also on the oocytes.

To further clarify the role of immune dysregulation in endometriosis-related infertility, the present study aims to investigate the impact of coexisting autoimmunity on embryo development and implantation in women affected by endometriosis. Understanding whether this association primarily affects the maternal interface (endometrium) or embryo development (oocyte) would provide an opportunity for translational research to optimize the clinical management of women seeking pregnancy. Therefore, we employed the in vitro fertilization–embryo transfer (IVF-ET) model, which allows for the observation of potential disease impacts on specific stages of the reproductive process including follicular growth, oocyte maturation, embryo development, and implantation.

## 2. Materials and Methods

### 2.1. Study Design and Patient Recruitment

This was a retrospective, multicenter case-control study that enrolled N = 600 women with endometriosis who underwent IVF-ET cycles between June 2007 and December 2021. The study was conducted at the tertiary care fertility units of two Centers: IRCSS San Raffaele Institute (Milan, Italy) and S. Anna Hospital and University (Turin, Italy). Cases were defined as women with endometriosis and concomitant autoimmunity, which was assessed through a medical interview at the first visit and confirmation of positive blood tests for autoantibodies. Cases were matched to controls in a 1:3 ratio by age (±6 months), body mass index (BMI) (±1 kg/m^2^), and year of IVF-ET cycle. The following 3 age- and BMI-matched women with endometriosis and without concomitant autoimmunity observed thus served as controls for each case. The inclusion criteria for enrollment were age ≥18 and ≤42 years, BMI ≥18.50 kg/m^2^ and ≤30 kg/m^2^, and a diagnosis of endometriosis confirmed either by surgery or transvaginal ultrasound (TVUS) finding of endometrioma and/or deep endometriosis lesions. Exclusion criteria included any other cause of couples’ infertility unrelated to endometriosis (i.e., male factor infertility), TVUS finding of adenomyosis, current major comorbidities other than autoimmunity requiring medication (i.e., hypertension), or poorly controlled autoimmune or inflammatory disease despite treatment. The study was conducted in accordance with the Declaration of Helsinki. Full Institutional Review Board (IRB) approval was not required due to the retrospective nature of the study. However, all patients undergoing IVF-ET gave informed consent for their anonymized data to be used for research purposes (protocol ENDOGIAWA1, approved on 14 October 2014).

### 2.2. IVF-ET Protocol

The protocols for IVF-ET cycles were as per the standard of clinical care, which is reported in detail elsewhere [18]. Briefly, the controlled ovarian stimulation (COS) regimen was initiated with recombinant follicle-stimulating hormone (FSH) on day 2–3 of the menstrual cycle, and the gonadotropin dose was individualized based on providers’ preference and patients’ characteristics. Follicular recruitment was monitored by serum progesterone and 17-beta-Estradiol (E_2_) and serial TVUS scans starting on or around cycle day 5–6 and repeated as necessary, usually on alternate days. A gonadotropin-releasing hormone (GnRH) antagonist at a daily dose of 0.25 mg was started when the leading follicle size reached about 12–14 mm. TVUS-guided oocyte retrieval was performed approximately 36–38 h after ovulation induction by human chorionic gonadotropin (5000 to 10,000 UI) or GnRH agonist (0.2–0.3 mg) administration. The decision for insemination (IVF) or intracytoplasmic sperm injection (ICSI) was based on clinical characteristics and sperm quality, as per the standard of clinical care. The resulting embryos were cultured and morphologically evaluated, and 1–2 embryos were transferred on day 3 or 5, under TVUS guidance. Vaginal progesterone at a dose of 200 mg three times daily was administered for luteal phase support and continued until approximately 9 weeks of pregnancy. In cases where a fresh ET did not establish pregnancy or when there was a clinical indication for a freeze-all strategy, frozen ET cycles were performed, utilizing exogenous oral estrogen (6 mg daily) and vaginal progesterone (800 mg daily) for endometrial preparation, according to the standard of care, and the outcomes of frozen embryo transfers were also recorded. A serum beta-HCG quantitative assessment was performed 14 days after fresh or frozen ET, and a serum level of >5 mIU/mL indicated evidence of implantation. A TVUS was performed at or beyond 7 weeks after ET, and a clinical pregnancy was diagnosed based on the presence of a normal intrauterine gestational sac with a presence of fetal cardiac activity. 

### 2.3. Data Collection and Study Outcomes

We reviewed medical records stored in electronic databases at the participating Centers. For all patients included in the study, we gathered the following data from their initial visit: age, BMI, type of infertility (primary or secondary, with data on previous pregnancy outcomes if applicable), medical history (past and present), history of previous pelvic surgery (including the year of surgery), localization of endometriosis lesions, presence of autoimmune disease (s), and basal ovarian reserve. The assessment of basal ovarian reserve included measuring the antral follicle count (AFC) and anti-Mullerian hormone (AMH) levels.

Consistent with a prior publication [19], we considered the following autoimmune diseases: autoimmune thyroid disorders (ATD), systemic lupus erythematosus (SLE), coeliac disease (CLD), multiple sclerosis (MS), Type 1 diabetes (T1D), Addison’s disease (AD), inflammatory bowel diseases (IBD), autoimmune hepatitis, Sjögren’s syndrome (SjS), undifferentiated connective tissue disease (UTCD), dermatomyositis (DM), systemic sclerosis (SS), psoriasis, rheumatoid arthritis (RA), vasculitis, and myasthenia gravis. The presence of Anti-Nuclear Antibodies (ANA) was also recorded. 

Endometriotic lesions were classified based on their localization as ovarian endometrioma (OMA), deep endometriosis (DE), and superficial peritoneal endometriosis (SPE) [20]. Expected poor ovarian responders were defined according to basal ovarian reserve biomarkers (AFC < 5 and/or AMH < 0.5 ng/mL) following the Bologna Criteria [21]. 

Data were prospectively recorded at the time of IVF-ET cycles and subsequently extracted and tabulated for analysis. We calculated the Ovarian Sensitivity Index (OSI) according to Huber et al. [22] as (number of retrieved oocytes/total FSH dose) × 1000. 

The primary outcome of the study was the cumulative clinical pregnancy rate (cCPR), defined as pregnancy resulting from both fresh and thawed cycles derived from a single egg collection procedure. Secondary outcomes were also calculated based on major international glossaries on Infertility care [23], including the metaphase II oocyte (MII) rate (no. MII oocytes/no. retrieved oocytes), fertilization rate (no. fertilized oocytes/no. inseminated oocytes), cleavage rate (no. embryos /no. fertilized oocytes), and implantation rate (no. gestational sacs/no. transferred embryos). 

### 2.4. Statistical Analysis

Statistical analysis was performed using STATA version 17 software (Stata Corp LLC, 2021, College Station, TX, USA). All tests were two-tailed, and *p* < 0.05 was judged as statistically significant. The normality of continuous variables was assessed using the Shapiro–Wilk test, along with skewness and kurtosis tests. Normally distributed continuous variables were presented as mean ± standard deviation (SD), while non-normally distributed continuous variables were expressed as median (Interquartile Range, IQR). Categorical variables were presented as absolute values and percentages (%). For the comparison of qualitative variables, we used Pearson’s chi-squared test or Fisher’s exact test, whereas, for quantitative variables, we used an independent Student’s *t*-test or Mann–Whitney U test based on the distribution of the variables. A sub-group analysis for the primary outcome based on basal ovarian reserve was further implemented. Finally, a logistic regression analysis was performed to identify independent predictors associated with cCPR. Only variables that were statistically significant at a threshold of *p*-value less than 0.05 in univariate analysis were tested in a multivariate-adjusted logistic regression model. Odds ratios (ORs), adjusted odds ratios (adjORs), and their corresponding 95% confidence intervals (95% CIs) were reported. The average marginal effects with 95% CIs were plotted in a graph of multiple regression models.

## 3. Results

### 3.1. Population Characteristics

A total of N = 600 women with endometriosis were enrolled: 150/600 (25%) had at least one concomitant autoimmune disease and were categorized as cases, while 450/600 (75%) were controls with endometriosis only. Among the recorded autoimmune diseases in the cases, ATD had the highest prevalence, equal to 101 out of 150 (67.33%). The baseline characteristics of the included patients are presented in Table 1. 

The mean age of cases and controls was 35.35 ± 3.95 and 35.86 ± 3.57 years, respectively, with no differences between the groups upon enrollment (*p* = 0.138). For most women in both groups, at least one surgery for endometriosis was performed before undergoing IVF-ET (88.89% in cases versus 90.60% in controls; *p* = 0.591), and no difference in the time from surgery to IVF-ET was observed (*p* = 0.700). The localization of endometriosis lesions was similar between cases and controls (*p* = 0.349): the majority of women in each group had an OMA or concomitant OMA and DE. At baseline, ovarian reserve markers were similar between cases and controls, with comparable total AFC [7.5 (5–12), vs. 7 (5–10), median (IQR), *p* = 0.385] and AMH values [1.38 (0.60–2.70) vs. 1.03 (0.50–2.04), median (IQR), *p* = 0.102]. In line, the proportion of expected poor responders according to the Bologna Criteria was similar between cases and controls (25.17% vs. 33.63, *p* = 0.060).

### 3.2. IVF Outcomes

IVF outcomes are presented in Table 2. No differences were observed in the FSH starting or total dose or the length of ovarian stimulation between the two groups (*p*> 0.05). Although the cases had a higher level of E2 on the day of hCG (1518 vs. 1304, *p* = 0.040) and a slightly higher median number of retrieved oocytes (5 vs. 4, *p* = 0.048) compared to controls, the similar OSI (*p =* 0.315) suggested no significant differences in ovarian response to COS between the two groups. Similarly, there were no differences in the proportion of MII oocytes (72.36% versus 73.26% in cases and controls, respectively; *p* = 0.386) and the fertilization rate (73.03% versus 73.26% in cases and controls, respectively; *p* = 0.252) between the two groups. However, the cleavage rate was significantly lower in cases (37.47%) compared to controls (43.98%; *p* = 0.042). The number of embryos transferred was similar in both groups (*p* = 0.438); however, the implantation rate was significantly lower in cases than in controls (10.73% versus 17.93%; *p* = 0.029). 

#### cCPR and Sub-Groups Analysis

**Figure 1:**
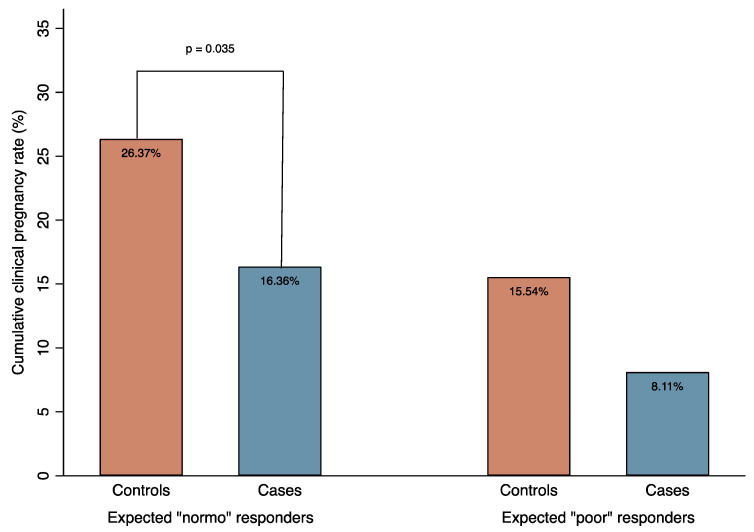
Cumulative clinical pregnancy rate (%) in cases and controls stratified by expected ovarian response.

The cCPR in cases reached 14.67%, which was significantly lower than in controls (22.44%; *p* = 0.041) (Table 2). In a sub-group analysis limited to women with a normal basal ovarian reserve (N = 402), the cCPR in cases was 16.36%, which was again significantly lower than in controls (26.37%; *p* = 0.035). Conversely, in the sub-group analysis performed on expected poor responders (N = 185), cCPR was not statistically different between the two groups (8.11% in cases versus 15.54% in controls; *p* = 0.301). Figure 1 shows the graph of cCPR in cases and controls stratified by expected ovarian response according to the Bologna Criteria [21].

### 3.3. Univariate and Multivariate Logistic Regression Analysis

**Figure 2:**
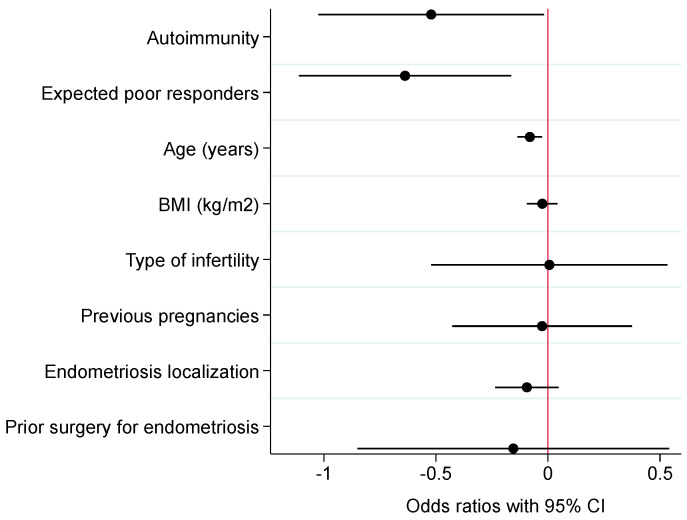
Average marginal effects with 95% confidence intervals (CI) of independent predictors associated with cumulative clinical pregnancy rate: univariate– and multivariate–adjusted models. Notes: black circles and solid lines represent, respectively, precise estimates and 95% CI under univariate analysis; navy circles and dashed lines represent, respectively, precise estimates and 95% CI under multivariate analysis; red solid vertical line is the null zero intercept. Abbreviations: BMI, body mass index.

The logistic regression analysis performed to assess independent predictors associated with cCPR is presented in Table 3. Among the clinically relevant variables tested, autoimmunity (*p* = 0.018), age (*p* = 0.007), and expected poor response (*p* = 0.014) were the only significant negative predictors of cCPR under both univariate and multivariate logistic regression analysis. In particular, the adjusted OR for autoimmunity under multivariate analysis was equal to 0.54 (95% CI 0.33–0.90). Figure 2 presents the average marginal effects with 95% CIs of independent predictors associated with cCPR under the univariate– and multivariate–adjusted models.

## 4. Discussion

### 4.1. Main Results

The results of the present study suggest that the co-occurrence of autoimmune diseases in women with endometriosis has a negative impact on their chances of achieving pregnancy. Women with endometriosis and autoimmune diseases exhibited lower rates of cleavage, implantation, and clinical pregnancy than controls with endometriosis alone. After adjusting for significant confounders in the multivariate model, the presence of autoimmunity in endometriosis reduced the odds of cCPR by 46% (adjOR 0.54; 95% CI 0.33–0.90; *p* = 0.018).

### 4.2. Comparison with the Existing Literature

The molecular mechanisms that lead to impaired implantation in women with endometriosis are still a matter of open debate. Particularly, it remains unclear whether this disease affects the implantation site impairing endometrial receptivity, embryo competence, or both. Some studies suggest that impaired implantation in endometriosis is solely due to a less-receptive eutopic endometrium [24]. Indeed, endometriosis-related chronic inflammation triggers several changes in the endometrial microenvironment and endometrial immune niche [25]. Molecular mechanisms, such as disrupted cell signaling pathways and decreased expression of essential homeostatic proteins, interact with each other, ultimately leading to local progesterone resistance and relative estrogen dominance [26]. Conversely, studies adopting the oocyte donation (OD) model have suggested that endometrial receptivity might not be impaired in women with endometriosis as long as a healthy embryo reaches the endometrial cavity [27]. According to this theory, endometriosis would impair oocyte quality and embryo development by causing damage related to intracellular reactive oxygen species (ROS) production [28]. The lower implantation rates observed in OD cycles where the donor has a history of endometriosis provide further support for this theory [29]. In addition, both local and systemic immune mechanisms that favor the growth and maintenance of endometriotic lesions may also play a role in endometriosis-related infertility, partly due to imbalanced immune cell populations and altered cytokine profiles in FF [30,31].

The lack of consensus regarding the relative contributions of the embryo and the endometrium to implantation processes in women with endometriosis may be due to the complexity of in vivo implantation networks and the difficulty of accurately reproducing them ex vivo [32]. However, it is plausible that these mechanisms are not mutually exclusive, as the process of human implantation is highly complex and dependent on the competence of the embryo, the receptivity of the endometrium, and the establishment of a finely tuned crosstalk between the two [12]. In women with endometriosis, both less-competent embryos [27,28] and less-receptive eutopic endometrium [24,25,26] may decrease the likelihood of successful implantation.

Similarly, autoimmune diseases have been associated with reduced MII oocytes and lower rates of fertilization, implantation, and clinical pregnancy due to increased levels of antibodies and cytokines in FF [33]. Some studies have linked the presence of ANA in FF, which correlates with serum ANA concentrations, to increased trophoblast apoptosis and decreased proliferation [34]. Additionally, ANA in FF may directly affect oocyte maturation and embryo formation, resulting in poorer IVF outcomes for women who are ANA seropositive compared to seronegative controls [35]. ANA positivity is more common in women with endometriosis, even in the absence of overt autoimmune disease, with an estimated prevalence of 30% in infertile women with endometriosis [36,37]. Notably, in our study cohort, we also found ANA positivity to be the second most common form of autoimmunity after ATD, which is the most prevalent type of autoimmunity in the general population, as well as in women affected by endometriosis and infertility [38].

In addition to autoantibody-mediated mechanisms, other immune-mediated factors that may contribute to infertility and impaired implantation in autoimmune diseases and endometriosis have been described. These factors include alterations in the balance of pro-inflammatory and anti-inflammatory cytokines, changes in the composition and/or activity of specific endometrial immune cells, and aberrant expression of adhesion molecules at the implantation site [39]. Recent studies have, in fact, shown that women with endometriosis present dysregulation of key endometrial receptivity-specific genes often belonging to immunological pathways [40]. A meta-analysis of the endometrial transcriptome has shown that women with endometriosis exhibit a pro-inflammatory profile characterized by increased levels of uterine natural killer (uNK) cells in the eutopic endometrium [41]. uNK cells are a subset of tissue-specific lymphocytes that play a critical role in embryo tolerance within the innate immune system. Dysregulation of these cells could affect the endometrium’s ability to support and allow embryo implantation [42]. Women with endometriosis also have a higher population of cytotoxic CD16+ uNK cells and/or NKp46+ CD56+ cells, which may contribute to an inflammatory microenvironment during decidualization [43]. Remarkably, the imbalance of uterine immune cells is also believed to contribute to impaired endometrial receptiveness in systemic autoimmune diseases [44]: aberrant expression of uNK CD56+ and CD16+ cells has been found in more than 80% of cases of refractory antiphospholipid antibody (APA)-mediated recurrent pregnancy loss [44].

**Figure 3:**
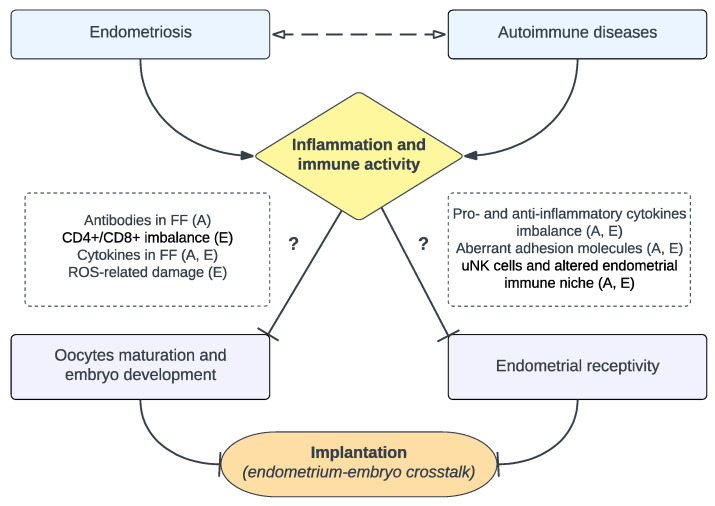
Overview of main mechanisms contributing to impaired implantation in endometriosis and autoimmune diseases. The presence of endometriosis or autoimmune diseases, or a combination of both, leads to increased inflammation and immune activity, which in turn might negatively impact both oocytes maturation/embryo development and endometrial receptivity, resulting in a disrupted endometrium–embryo crosstalk at the implantation site. Immunological and inflammatory mechanisms implicated in endometriosis (E) and autoimmune diseases (A) are outlined in dashed boxes. Abbreviations: FF, follicular fluid; ROS, reactive oxygen species; uNK, uterine natural killer.

To summarize, the exact role of inflammatory and immunological changes in endometriosis-related infertility remains unclear [45]. Understanding the immune system in reproduction is a challenging task due to the complexity of the immunology at the maternal–fetal interface, which involves various molecular, cellular, tissutal, and systemic processes and interactions [46]. However, the presence of shared mechanisms that affect embryo development and endometrial receptiveness in both endometriosis and autoimmune diseases (Figure 3) suggests that the embryo–endometrial crosstalk may be even more dysfunctional when these diseases occur together.

### 4.3. Strengths and Limitations

This study has several notable strengths. First, it is the first study to investigate the early reproductive steps of women affected by both autoimmunity and endometriosis, compared to controls with endometriosis only. The high biological plausibility justifying the association found, coupled with the common incidence of both endometriosis and autoimmunity, underscores the high significance of our study. Second, the multicenter nature of the study enabled the enrollment of a relatively large cohort of women, which enhances the reliability and generalizability of the results. Third, the exclusion of patients with adenomyosis from the cohort ensures that our final estimates are more consistent for the effect of endometriosis alone, avoiding any potential disruptive effects of adenomyosis on the myometrium and endometrium. Adenomyosis is a significant confounder when assessing reproductive outcomes in endometriosis because of its well-known independent impact on implantation [47]. Additionally, the sub-group analysis conducted according to the Bologna Criteria for expected poor responders [21] increases the reproducibility of our results in specific cohorts of endometriosis-related infertile women and other clinical settings.

It is important to note some limitations of this study. First, the study’s retrospective design may have affected the collection of data, despite the use of prospectively collected electronic databases during IVF-ET cycles. Additionally, while a laparoscopic diagnosis was present for most (>85%) of cases, the fact that some patients included had a U.S.-based diagnosis of deep and ovarian endometriosis can be considered as a limitation. However, TVUS assessment for deep and/or ovarian endometriosis has been validated as a reliable diagnostic tool when conducted in tertiary clinical settings [48]. Another limitation was the lack of information on the stage of endometriosis in the cohort. Due to unavailable surgical reports during data extraction and analysis, the calculation and reporting of surgical scores for disease severity were inaccurate. Nevertheless, since the localization of endometriotic lesions and the proportion of surgically treated women prior to IVF-ET were similar in both comparison groups, it is plausible that the disease’s severity was similar as well. A further limitation was the unavailability of data on autoantibody titers, limited by missing data and variability between laboratories. Further studies examining endometriosis severity and autoantibody titers are strongly encouraged to better understand the potential correlation between disease severity and implantation failure and to confirm the current findings.

Furthermore, in the present study, information on oocyte quality was not available, and there was no dynamic evaluation of embryo development. Morphologic evaluation of embryo quality is influenced by both inter- and intra-operator variability, which limits its effectiveness in identifying the most competent embryos [49]. Future studies could potentially use incubators with integrated time-lapse monitoring systems to analyze specific embryo developmental steps that may be impacted in these patients. It should be noted that the study did not report data on live births, as this was not the primary endpoint. The evaluation was limited to the impact of coexisting autoimmunity and endometriosis on the early phases of embryo development and implantation. Further research is necessary to investigate the effect of autoimmune diseases in endometriosis on later pregnancy outcomes, including perinatal outcomes, as well as maternal and fetal morbidities.

## 5. Conclusions

This study highlights the detrimental impact of autoimmune diseases on reproductive outcomes in women with endometriosis. Using the IVF model, we have demonstrated that the co-occurrence of these diseases compromises both embryo development and implantation. These findings indicate that clinicians should be aware of the potential lower chances of pregnancy in the presence of autoimmune comorbidities when counseling infertile women with endometriosis. In such cases, a timely and effective intervention to reduce the overall inflammatory status could be recommended as a possible prevention strategy.

## Figures and Tables

**Table 1:** Baseline characteristics (N = 600).

	Cases (*n* = 150)	Controls (*n* = 450)	*p*-Value
Age (years)	35.35 ± 3.95	35.86 ± 3.57	0.138
BMI (kg/m^2^)	21.87 [20.00–23.73]	21.26 [19.75–24.00]	0.370
Type of infertility			0.380
Primary	121 (80.67%)	377 (83.78%)	
Secondary	29 (19.33%)	73 (16.22%)	
Previous pregnancies			0.703
None	124 (82.67%)	376 (83.56%)	
Miscarriage	18 (12%)	57 (12.67%)	
Term pregnancy	8 (5.33%)	17 (3.78%)	
Endometriosis localization			
OMA	74 (49.33%)	206 (45.78%)	0.349
DE	10 (6.67%)	49 (10.89%)	
OMA + DE	31 (20.67%)	111 (24.67%)	
SPE	11 (7.33%)	24 (5.33%)	
Unknown	24 (16%)	60 (13.33%)	
Prior surgery for endometriosis	104 (88.89%)	251 (90.60%)	0.591
Time from surgery to IVF (months)	24 [12–72]	24 [12–72]	0.700
Type of autoimmunity		450 (100%)	**0.000**
None			
ATD ^1^	101 (67.33%) ^2^		
SLE	7 (4.67%)		
CLD ^1^	4 (2.67%)		
MS	4 (2.67%)		
T1D ^1^	2 (1.33%)		
Psoriasis	1 (0.67%)		
IBD	4 (2.67%)		
SjS	1 (0.67%)		
UTCD	5 (3.33%)		
DM	2 (1.33%)		
SS	1 (0.67%)		
ANA positivity	20 (13.33%)		
AMH (ng/mL)	1.38 [0.60–2.70]	1.03 [0.50–2.04]	0.102
AFC	7.5 [5–12]	7 [5–10]	0.385
Expected poor responders ^3^	37/147 (25.17%) ^4^	148/440 (33.63%) ^4^	0.060

Notes: data are reported as mean (standard deviation) or median [25–75%] or n (%). Abbreviations: COS, controlled ovarian stimulation; BMI, Body mass index; ART, assisted reproductive techniques; ATD, Autoimmune thyroid disorders; SLE, Systemic Lupus Erythematosus; CLD, Coeliac disease; MS, Multiple Sclerosis; T1D, Type 1 diabetes; IBD, inflammatory bowel diseases; SjS, Sjögren’s syndrome; UTCD, undifferentiated connective tissue disease; DM, Dermatomyositis; SS, Systemic Sclerosis; ANA, Anti-Nuclear Antibodies; AMH, Anti-mullerian hormone; AFC, antral follicle count. ^1^ One patient with CLD and one patient with T1D carried also ATD. ^2^ Total number of patients with ATD, including the two cases with more than one concomitant autoimmune disease. ^3^ Defined from basal ovarian reserve markers according to the Bologna Criteria [21] as AMH < 0.5 ng/mL and/or AFC < 5. ^4^ For 3 cases and 10 controls, data on ovarian reserve were not available.

**Table 2:** IVF outcomes.

	Cases (*n* = 150)	Controls (*n* = 450)	*p*-Value
FSH starting dose (UI)	254.28 ± 84.38	267.41 ± 76.72	0.166
FSH total dose (UI)	2837.5 [2025–3900]	3000 [803–2008]	0.395
Length of stimulation (days)	11 [9–13]	11 [9–12]	0.301
E_2_ on day of hCG, pg/mL	1518 [871–2391]	1304 [2250–3900]	**0.040**
No. oocytes retrieved	5 [2–10]	4 [2–7]	**0.048**
Unsuccessful OPU, %	8% (12/150)	8.67% (39/450)	0.800
OSI ^1^	1.78 [0.74–3.85]	1.63 [0.73–3]	0.315
MII oocytes	4 [2–8]	3 [2–6]	0.067
MI oocytes	1 [0–2]	0 [0–1]	0.182
MII rate ^2^, %	72.36% (699/966)	73.26% (1745/2382)	0.386
Fertilization rate ^2^, %	73.03% (547/749)	68.89% (1280/1858)	0.253
Cleavage rate ^2^, %	37.47% (205/547)	43.98% (563/1280)	**0.042**
No. embryos transferred	2 [1–2]	1 [1–2]	0.438
ET fresh cycles, %	59.33% (89/150)	64.00% (288/450)	0.306
Implantation rate ^2^, %	10.73% (22/205)	17.93% (101/563)	**0.029**
cCPR, %	14.67% (22/150)	22.44% (101/450)	**0.041**

Notes: data are reported as mean (standard deviation) or median [25–75%] or % (ratio). Abbreviations: FSH, follicle-stimulating hormone; E_2_, serum estradiol; hCG, human Chorionic Gonadotropin; OPU, oocytes pick-up; OSI, ovarian sensitivity index; MII, metaphase II; MI, metaphase I; ET, embryo transfer; cCPR, cumulative clinical pregnancy rate. ^1^ Calculated as: no. retrieved oocytes/FSH total dose × 1000. ^2^ Calculated as follows: MII rate: (no. MII oocytes/no. retrieved oocytes); fertilization rate: (no. fertilized oocytes/no. inseminated oocytes); cleavage rate: (no. embryos/no. fertilized oocytes); implantation rate: (no. gestational sacs/no. transferred embryos).

**Table 3:** Logistic regression models of independent predictors associated with cumulative clinical pregnancy rate.

	Univariate Logistic Regression			Multivariate Logistic Regression		
Parameters	OR	95% CI	*p*-Value	AdjOR	95% CI	*p*-Value
Autoimmunity	0.59	0.36–0.98	**0.043**	0.54	0.33–0.90	**0.018**
Expected poor response	0.53	0.33–0.86	**0.010**	0.56	0.34–0.90	**0.017**
Age (years)	0.92	0.87–0.97	**0.004**	0.93	0.87–0.98	**0.007**
BMI (kg/m^2^)	0.97	0.91–1.04	0.469			
Type of infertility	1.01	0.59–1.70	0.981			
Previous pregnancies	0.97	0.65–1.45	0.899			
Endometriosis localization	0.91	0.79–1.05	0.193			
Prior surgery for endometriosis	0.86	0.43–1.71	0.664			

## Data Availability

The data that support the findings of this study are available from the first author (N.S.) or the corresponding author (V.S.V.), upon reasonable request.
